# Oyster Shell Proteins Originate from Multiple Organs and Their Probable Transport Pathway to the Shell Formation Front

**DOI:** 10.1371/journal.pone.0066522

**Published:** 2013-06-19

**Authors:** Xiaotong Wang, Li Li, Yabing Zhu, Yishuai Du, Xiaorui Song, Yuanxin Chen, Ronglian Huang, Huayong Que, Xiaodong Fang, Guofan Zhang

**Affiliations:** 1 Institute of Oceanology, Chinese Academy of Sciences, Qingdao, China; 2 BGI-Shenzhen, Shenzhen, China; Georg August University of Göttingen, Germany

## Abstract

Mollusk shell is one kind of potential biomaterial, but its vague mineralization mechanism hinders its further application. Mollusk shell matrix proteins are important functional components that are embedded in the shell, which play important roles in shell formation. The proteome of the oyster shell had been determined based on the oyster genome sequence by our group and gives the chance for further deep study in this area. The classical model of shell formation posits that the shell proteins are mantle-secreted. But, in this study, we further analyzed the shell proteome data in combination with organ transcriptome data and we found that the shell proteins may be produced by multiple organs though the mantle is still the most important organ for shell formation. To identify the transport pathways of these shell proteins not in classical model of shell formation, we conducted a shell damage experiment and we determined the shell-related gene set to identify the possible transport pathways from multiple organs to the shell formation front. We also found that there may exist a remodeling mechanism in the process of shell formation. Based on these results along with some published results, we proposed a new immature model, which will help us think about the mechanism of shell formation in a different way.

## Introduction

Shells are one of the most remarkable characteristics of mollusks and they provide interesting insights in many research fields. Some molluscan shell proteins can affect bone resorption and these may be shared by vertebrate and mollusk in the process of mineralization, and nacre is sustained when implanted into bone tissue and thus it is one kind of potential bone substitute and other biomaterials [1], [2], [3], [4]. But, the biomineralization mechanism of shell formation still remains unclear, which hinders the application of shell products in the area of biomaterials.

DNA has been isolated from the oyster and other mollusk shells [5], [6], which suggests that active and complex life processes existed in these mineralized tissues during their formation. The identification of shell matrix proteins (SMPs) is very important for clarifying the mechanism of shell formation [7]. The availability of the oyster genome makes it possible to identify SMPs on a large scale. Our group has determined the proteome of shell proteins [8] and found that most of oyster shell proteins are nonsecretory and cells may be involved in shell formation, also hinting the complexity of shell formation. In addition, the phenomenon of shell dissolution has been observed [9], [10] in previous studies though the molecular basis remains unclear, which the shell is an active and complex tissue and can change after formed. The classical model of shell formation posits that mineralization occurs in a mantle-secreted matrix of chitin, silk fibroin and acidic proteins, i.e., the shell proteins are produced by mantle and secretory [11]. Obviously, new discoveries make us reconsider the model of shell formation.

In this study, after further analyzing shell proteome data in combination with organ transcriptome data, we found that shell proteins may originate from multiple organs, rather than the mantle alone; after GO enrichment analysis on the proteome data of oyster shell, we found that the probable molecular mechanism for shell dissolution; to identify the genes involved with the shell formation process, we investigated genes that are differentially expressed in the mantle in response to shell damage; we also found that several pathways may be involved in the transport of shell proteins originating from multiple organs. At last, based on these results along with some published results, we tried to propose a new model for shell formation.

## Materials and Methods

### Animals

The Pacific oyster whose genome was sequenced is an inbred female produced by four generations of brother–sister mating. She was provide by Pro. Dennis Hedgecock in University of Southern California, USA, and were cultured in the aquarium at Institution of Oceanology, Chinese Academy of Sciences (IOCAS). The Pacific oysters used to extract the shell proteins, to sequence the transcriptomes of seven organs or to carry out the shell damage experiment are two-year old and 9–12 cm in shell length. They were purchased from a farm in Weihai, China, and were cultured in the aquarium at IOCAS.

### Identification of shell protein genes

The sequencing of the oyster genome offers a unique opportunity for studying molluscan shell formation. In previous study, we isolated proteins, both soluble and insoluble, from oyster shells, obtained peptidase sequences and used them to identify 259 shell protein genes in the oyster genome [8]. The standard of protein identification is as follows: the unique peptides had a length of at least 6 aa peptide. Peptide sequences were searched against the oyster protein set using Mascot software. Only when the peptide matches a unique gene and the score is greater than or equal to mascot identity score, the protein is considered as identified in the oyster shell.

### The process of RNA-seq experiment

Total RNA was extracted using the guanidinium thiocyanate-phenol-chloroform extraction method (Trizol, Invitrogen) according to manufacturer’s protocol. Poly-A RNA was isolated with oligo-dT-coupled beads from 20 µg total RNA of each sample and then sheared, and the isolated RNA samples were used for first strand cDNA synthesis which was performed with random hexamers and Superscript II reverse transcriptase (Invitrogen). The second strand was synthesized with *E. coli* DNA PolI (Invitrogen). Double stranded cDNA was purified with Qiaquick PCR purification kit (Qiagen, Germantown, MD). After end repair and addition of a 3’ dA overhang, the cDNA was ligated to Illumina paired-end adapter oligo mix, and size selected to about 200 bp fragments by gel purification. After 15 PCR cycles the libraries were sequenced using Illumina sequencing platform and the paired-end sequencing module.

### Organ distribution of shell protein genes

The transcriptomes of seven organs, i.e., the mantle, digestive gland, gill, adductor muscle, hemolymph, labial palps, female and male gonad, were acquired from one individual (except the male gonad from another individual) using 90 bp paired-end RNA-seq [8]. The gene expression levels of shell proteins were determined in different organs based on their RPKM values (reads per kilobase of gene model per million mapped reads). The number of expressed shell protein genes (at least RPKM > 5) were recorded in each organ.

### GO enrichment analysis of the shell matrix proteins

Based on functional annotation using Gene Ontology (GO), we extracted GO terms that were enriched in shell proteins with EnrichPipeline [12].

### Shell damage treatment

Experimental oysters were divided into eight groups, including one control group and seven treated groups that sustained shell damage for 1, 3, 5, 9, 13, 17, and 21 days, with six individuals in each group. To produce shell damage, part of the left side of the shell was carefully removed using an electric drill with flat sanding head, without damaging the mantle.

### Gene expression profiles of the left and right mantles

On each sampling date, the left and right mantles were collected from each oyster and their total RNAs were extracted separately. The total RNAs from the left mantles of six individuals in the same group were combined equally to produce sample for 49 bp single-end RNA-seq analysis, and the same method was applied to the right mantles. The numbers of upregulated and downregulated genes in the right and left mantles compared with the control were counted in samples collected on each of the seven sampling dates (*P*<10^−3^).

### Construction of a new model of shell formation

A new model for shell formation was proposed based on a combination of the organ distribution of shell proteins, the functional properties of shell proteins, the pathways probably involved in shell formation and some published results.

## Results and Discussion

### Multiple-organ distribution of shell protein genes

**Figure 1:**
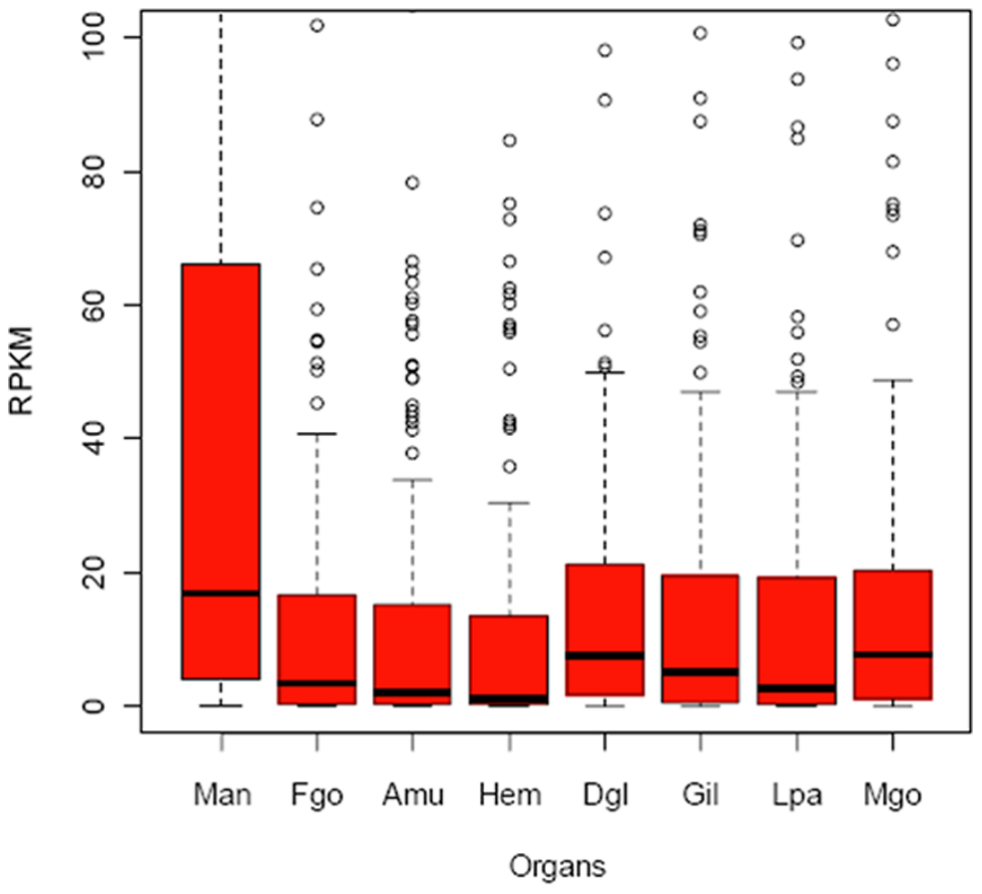
The gene expression level of shell proteins in the mantle was the highest among all organs. The *y*-axis represents the RPKM values of shell protein genes, while the *x*-axis shows the different organs. Mantle is abbreviated as Man, female gonad, Fgo; adductor muscle, Amu; hemolymph, Hem; digestive gland, Dgl; gill, Gil; labial palps, Lpa; male gonad, Mgo. The total expression level of shell proteins in the mantle was the highest, but there were also highly expressed genes in other organs.

**Figure 2:**
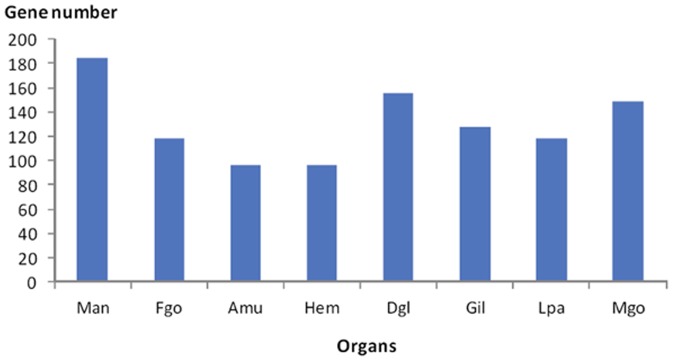
The number of expressed shell protein genes in the eight organs. The *y*-axis shows the number of expressed shell protein genes (RPKM > 5), while the *x*-axis shows the different organs. Mantle is abbreviated as Man, female gonad, Fgo; adductor muscle, Amu; hemolymph, Hem; digestive gland, Dgl; gill, Gil; labial palps, Lpa; male gonad, Mgo.

The highest expression level of shell protein genes in mantle suggested that the mantle was the main organ involved in shell formation (Fig. 1), in accord with the classical model of shell formation. However, most of the identified shell protein genes were expressed widely in all organs (Fig. 2), suggesting the multi-organ origin of most shell proteins. Interestingly, the 27 genes that were not expressed in the mantle (RPKM < 5 was treated as unexpressed) had at least twofold expression in other organs (RPKM > 5) (Table S1), which suggested that some shell proteins may be produced by organs other than the mantle. In addition, the 22 SMP genes that were not expressed in the mantle or the hemolymph had at least twofold expression in other organs (RPKM > 5) (Table S2), which further suggested that shell proteins may also be produced by organs outside the mantle and the hemolymph [13]. More importantly, the observation expelled probability of the hemocytes distributed in other organs causing the multiple-organ distribution of shell proteins.

Osteopontin, a prominent component of the mineralized extracellular matrices of bones and teeth, is biosynthesized by a variety of tissue types [14]. Similarly, a 48 kDa shell matrix protein has been detected in multiple organs [15]. Our results further suggest that the multiple-organ distribution of biomineralization proteins may be ubiquitous in species with mineralized tissues. The shell protein genes not expressed in the mantle or the hemolymph but in other organs suggested that these shell protiens may be transported to the shell formation front through some particular pathways.

### GO enrichment analysis for shell proteins suggests the shell remodeling

The GO enrichment results for oyster shell proteins are shown in Table 1, some of which are considered to be related to the decomposition and construction of the shell organic framework, such as chitin binding and chitin metabolic process, because chitin is one of the important components of shell organic framework [11]. Therefore, there may be a remodeling mechanism during the process of shell formation, just as the bone remodeling [16]. In this progress, some parts of the old shell is catabolized and reconstructed to join with the new shell when the new shell is forming. Importantly, the remodeling of shell inorganic component, calcite crystals, has been reported in the Eastern oyster shell [17] and the shell proteins should regulate the process. Therefore, the GO terms related to chitin binding and metabolism enriched in shell proteins suggested that the shell organic framework could be reconstructed during the process of shell formation, which might ensure a tight linkage between the old and new shell. The phenomena of shell dissolution [9], [10] suggested the existence of shell remodeling in mollusks. The GO enrichment results may give a molecular support to the phenomena of shell dissolution.

**Table 1:** GO enrichment results for oyster shell proteins.

GO_ID	GO_Term	GO_Class	*P*-value
GO:0009308	amine metabolic process	BP	0.000149
GO:0006022	aminoglycan metabolic process	BP	7.26E-07
GO:0030246	carbohydrate binding	MF	3.77E-05
GO:0005975	carbohydrate metabolic process	BP	5.98E-06
GO:0008061	chitin binding	MF	8.90E-06
GO:0006030	chitin metabolic process	BP	1.98E-06
GO:0004866	endopeptidase inhibitor activity	MF	6.69E-07
GO:0030414	peptidase inhibitor activity	MF	5.83E-10
GO:0004867	serine-type endopeptidase inhibitor activity	MF	1.83E-05
GO:0030234	enzyme regulator activity	MF	5.22E-06
GO:0005576	extracellular region	CC	6.02E-08

### Identification of the genes related to shell formation

The numbers of upregulated and downregulated genes in the right and left mantles compared with the control right and left mantles were counted in samples collected on each of the seven sampling dates (*P*<10^−3^). We selected genes that were upregulated in the left mantle on at least six of the seven sampling dates as the genes corresponding to shell damage. This strategy was based on an assumption that the number of upregulated genes on one sampling date should be highest whereas those on all seven sampling dates should be lowest, so the decrease should follow a gradual random distribution if the expression of genes was not affected by shell damage.

**Figure 3:**
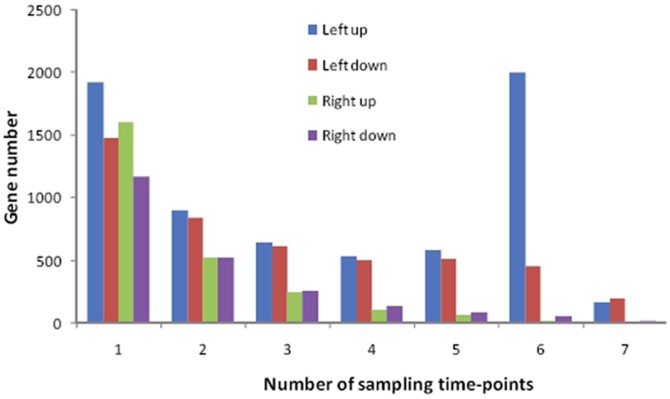
The numbers of upregulated and downregulated genes in the right and left mantles compared with the control right and left mantles on each of the seven sampling dates. In the right mantle, the number of upregulated or downregulated genes (green and purple bars, respectively) were determined at 1, 2, 3, 4, 5, 6 and 7 sampling dates. The levels gradually decreased with the increase of sampling dates, as expected if upregulation or downregulation were caused by random variation. So is the downregulated genes in left mantle (red bar). However, the distribution of upregulated genes was not random in the left mantle (blue bar), and abnormally large numbers of genes were upregulated on six sampling dates, which were probably due to shell damage. Thus, the genes upregulated on 6/7 sampling dates in the left mantle were treated as candidate genes that responded to shell damage (i.e., the gene set related with shell formation).

As expected, the gene numbers were randomly distributed in the right mantle, and the distribution of downregulated genes was also random in the left mantle (Fig. 3). However, the abnormally large number of upregulated genes on the six sampling dates was probably due to shell damage, which suggested that the data were credible. Thus, the genes that were upregulated on 6/7 sampling dates in the left mantle were selected as the genes related to shell formation.

In addition, only the left mantle responded to left shell damage, which suggests that the left and right mantles are functionally separated, at least in terms of their biomineralization function, though they are linked as one whole.

### Functional analysis of the genes related to shell formation

We detected 2174 upregulated genes after the shell damage treatment and their corresponding proteins were annotated as multifunctional (Table S3; *p*<e^−5^), suggesting that many genes and pathways were involved in shell formation, rather than only the shell proteins embedded in the shell alone. In addition, only 33 of 259 shell proteins were found upregulated after shell damage in mantle (Table S4), suggesting that mantle may not be the only organ involved in shell formation, which is consistent with the above deduction that the shell proteins were produced by multiple organs.

Some well-known shell formation-related genes were clearly upregulated after shell damage, including *PFMG4* (CGI_10005220), *Pif177* (CGI_10014497), *dermatopontin* (CGI_10024233), and *tyrosinase* (CGI_10017214) (Fig. S1). Their upregulation provided strong evidence for their involvement in shell formation. *PFMGs* are highly expressed in the mantle of the pearl oyster *Pinctada fucata* and they may play important roles in nacre biomineralization [18]. Because there is no nacre in oyster shell, *PFMGs* may act similarly in oyster calcite shell. *Dermatopontins* are EMC proteins that may play a role in shell matrix formation in the same way as *fibronectin* [8], [19].

The function of upregulated genes was highly diverse and clearly not limited to shell formation. *GPCR*, *ghC1qDC* gene, and *c-type lectins* were among the highly upregulated genes, as were many members of the *epidermal growth factor* family, *lipid transport protein,s* and *ATPases*. Many proteins may participate directly and indirectly in shell formation and constitute a complex gene network during shell formation.

### Pathway enrichment of the genes related to shell formation

There were 55 enriched KEGG pathways (Table S5; *p*<0.05) when analyzing the upregulated genes after the shell damage treatment. Interestingly, the chemokine signaling pathway and leukocyte transendothelial migration pathways were therein. This finding, along with the report that granulocytic hemocytes, a type of leukocyte, may transport calcite crystals [13], hinted that shell proteins not produced by the mantle could be discharged into the hemolymph and swallowed by granulocytes therein; next, granulocytes containing these shell proteins (maybe also calcium carbonate crystals) were targeted by chemokines to the mantle and then transendothelially migrated through the mantle membrane; finally, they arrived at the shell mineralization front, where they burst open to release crystals and these shell proteins. So, the shell proteins not produced by mantle got to shell formation front.

### A new model of shell formation

**Figure 4:**
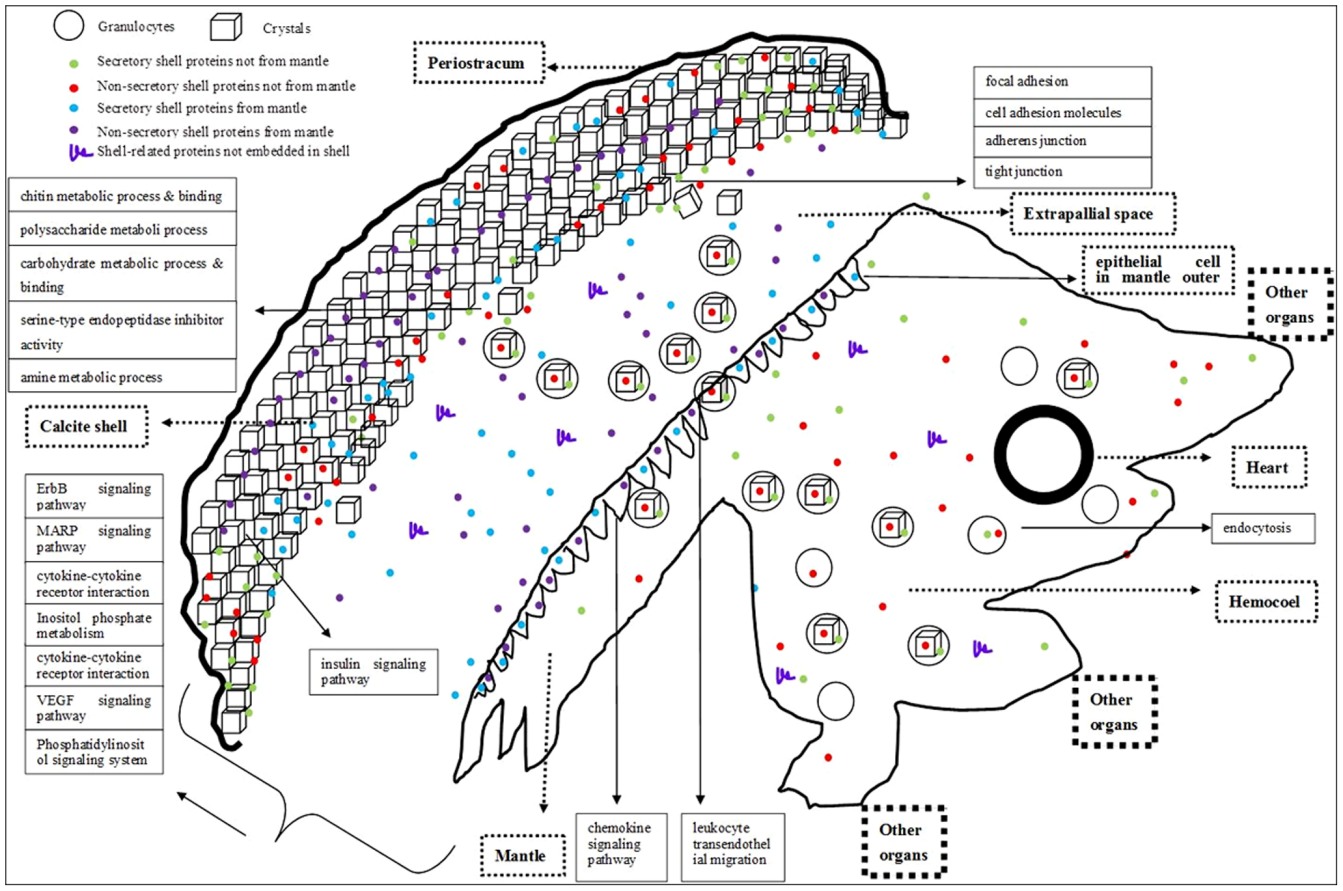
A new molecular model for oyster shell formation. The notes for some of the shapes included in this figure are shown on the upper left. In general, the figure is divided into two parts: soft body and shell. The soft body including all the organic organs is represented as polygon (bottom-right), whereas the shell including the formed shell, forming shell, and periostracum (top-left) is represented as the large arc and some cubes. The large thick circle denotes the heart. All of the oyster organs surround the common hemolymph pool (hemocoel) and they also make contact with each other. Some shell proteins produced by organs other than the mantle (i.e., the shell proteins not produced by the mantle) may arrive into the hemocoel firstly and then be engulfed by the granulocytes.

Based on our results and previous publications, we tried to propose a new model for oyster shell formation (Fig. 4). Depending on the production site and whether proteins are secretory or nonsecretory, shell proteins can be divided into the following four categories: a. secretory shell proteins not produced by the mantle; b. nonsecretory shell proteins not produced by the mantle; c. secretory shell proteins produced by the mantle; d. nonsecretory shell proteins produced by the mantle. The shell proteins from categories c and d will directly be transported to the biomineralization front from mantle via exosome [8] or classical secretory pathway. The mantle is still the most important organ for shell formation. The shell proteins from categories a and b are firstly transported to the hemolymph via classical (for secretory) and nonclassical secretory pathways [20] (for nonsecretory) from other organs, then engulfed by granulocytes (one kind of hemocyte). Granulocytes containing calcium carbonate crystals and shell proteins engulfed or produced by themselves are piloted by chemokines in the mantle, after get to the mantle, they migrate transendothelially through the mantle to the shell formation front (supported by the enriched pathways based on the genes related to shell formation: chemokine signaling pathway and eukocyte transendothelial migration). The granulocytes then disintegrate and release shell proteins, which are not produced by the mantle, and calcium carbonate crystals there. Together with the shell proteins made by the mantle, they interact with each other, or with polysaccharide or chitin, to produce the shell framework. Remodeling may occur during the process of shell growth, which is supported by the enrichment of the GO terms related to chitin binding and metabolism in shell proteins (Table 1). Shell formation may be regulated by many signal pathways, such as the insulin signaling pathway (the known biomineralization-related pathway), as well as the ErbB signaling pathway and MAPK signaling pathway.

## Conclusions

Our novel findings that the multiple-organ origin of shell proteins, the probable proteins related with shell remodeling, the diverse functions of the genes related with shell formation and the possible pathways for shell protein transport suggest the presence of complex life processes when shell forming, which will stimulate more detailed research in this area. In the future, many physiological experiments should be performed to prove the immature model proposed in this study.

## Supporting Information

Figure S1 **Upregulation of oyster homologues of known shell formation-related genes after shell damage.** The left *y*-axis represents the expression in RPKM units of *PFMG*, *Pif177,* and *Dermatopontin*, whereas the right *y*-axis represents the expression of *Tyrosinase*, The *x*-axis represents the number of days after shell damage.
(PDF)

Table S1 **The 27 SMPs not expressed in the mantle but in other organs.**
(XLSX)

Table S2 **The 22 SMPs not expressed in the mantle or hemocytes but in other organs.**
(XLSX)

Table S3 **Genes upregulated on 6 and 7 sampling dates in the left mantle after shell damage.**
(XLSX)

Table S4 **The 33 shell proteins identified in the shell proteome and also upregulated on 6 and 7 sampling dates in the left mantle after shell damage.**
(XLSX)

Table S5 **The KEGG enrichment results for the genes that were upregulated on 6 and 7 sampling dates in the left mantle after shell damage.**
(XLSX)
