# Supplementary figures and images for: Oyster Shell Proteins Originate from Multiple Organs and Their Probable Transport Pathway to the Shell Formation Front
Source: PLoS One. 2013 Jun 19;8(6):e66522. doi: 10.1371/journal.pone.0066522 (PMC3686672; doi:10.1371/journal.pone.0066522)

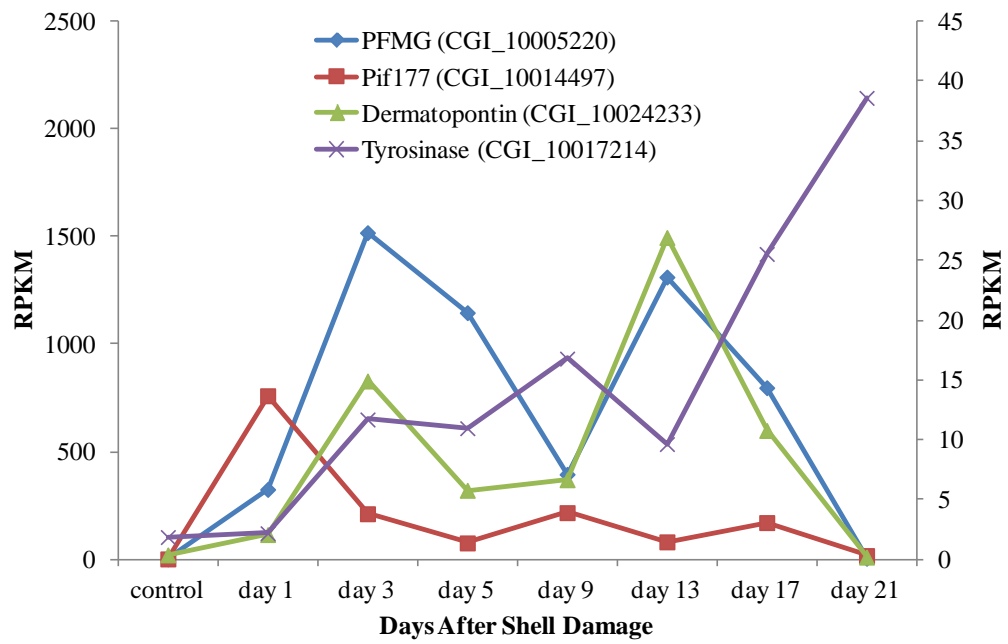

Supplement: Figure S1 — Upregulation of oyster homologues of known shell formation-related genes after shell damage. The left y-axis represents the expression in RPKM units of PFMG, Pif177, and Dermatopontin, whereas the right y-axis represents the expression of Tyrosinase, The x-axis represents the number of days after shell damage. (PDF) [file pone.0066522.s001.pdf]
